# Machine Learning‐Assisted Optimization of Iodide Electrolytes for Efficient Indoor Dye‐Sensitized Solar Cells with Engineered Photoanodes

**DOI:** 10.1002/smsc.70324

**Published:** 2026-06-13

**Authors:** Valid Mwalukuku, Antonio R. Blanco, Cyril Aumaître, Yann Kervella, Said Hamad, Renaud Demadrille, Juan A. Anta

**Affiliations:** ^1^ Center for Nanoscience and Sustainable Technologies (CNATS) Department of Physical, Chemical, and Natural Systems Universidad Pablo de Olavide Sevilla Spain; ^2^ IRIG‐SyMMES CEA CNRS Grenoble INP Université Grenoble Alpes Grenoble France

**Keywords:** dye‐sensitized solar cells, impedance spectroscopy, indoor photovoltaics, machine learning

## Abstract

Although dye‐sensitized solar cells (DSSCs) have lower power conversion efficiencies than other emerging photovoltaic technologies under standard test conditions, they perform remarkably well under low light or artificial lighting. This makes them particularly well suited to indoor photovoltaics (IPVs) and powering Internet of Things (IoT) devices. The most efficient indoor DSSCs rely on copper‐based redox shuttles to achieve high photovoltages; however, their long‐term stability is still uncertain. In contrast, iodide‐based electrolytes are robust under harsh and outdoor conditions, but under indoor illumination, they often deliver limited photovoltage. Here, we propose a strategy that combines an innovative photoanode architecture, designed to suppress charge recombination, with a design of experiments (DoE) approach that is integrated with machine learning (ML). This strategy is intended to optimize iodine‐based electrolytes for high‐performance IPVs. Support vector machines and Bayesian optimization were employed to accelerate development, achieving efficiencies surpassing 23% at 1000 Lx. As photovoltage is crucial for IoT applications, it was specifically targeted during ML optimization, achieving up to 683 mV at 500 Lx using an iodide/triiodide redox couple. Bayesian optimization also enabled the exploration of electrolyte formulation, achieving 713 mV at 1000 Lx. These results are among the highest reported for iodide/triiodide‐based DSSCs under indoor lighting.

## Graphical abstract

We developed dye‐sensitized solar cells (DSSCs) featuring an innovative photoanode architecture and employed a design‐of‐experiments (DoE) integrated with machine learning (ML) approaches to optimize iodide/triiodide‐based electrolytes for indoor photovoltaics. This strategy delivered open‐circuit voltages exceeding 0.71 V and power conversion efficiencies above 23% at 1000 Lx.

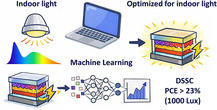

## 1 Introduction

Dye‐sensitized solar cells (DSSCs) are emerging as the preferred photovoltaic technology for powering indoor devices, thanks to the low‐cost of their manufacturing process and relatively high efficiencies under standard and diffuse illumination conditions [1, 2]. Their scalability opens possibility of up‐scaling in small modules and they can be incorporated into flexible substrates thus facilitating their integration in Internet of Things (IoTs) devices in domestic environments [3, 4]. Among emerging and mature technologies such as Silicon and gallium arsenide (GaAs), DSSCs demonstrate exemplary performance, bandgap versatility, semitransparency [5, 6], and ease of processing with low carbon footprint and toxicity levels [7]. Consequently, they have attracted significant interest from both academia and industry toward powering connected indoor devices. DSSCs operate under standard AM 1.5G conditions with power conversion efficiencies (PCE) exceeding 15% [8]. DSSCs have three major components: a photoanode, composed of a mesoporous TiO_2_ layer covered by a photosensitizer, a photocathode functionalized with Pt, and an electrolyte connecting the two electrodes. The design and working principles under standard AM 1.5G conditions have been widely investigated and defined. However, a great deal of work is still needed to optimize the performance of low‐light DSSC applications.

For example, under outdoor conditions the photosensitizer must demonstrate extended absorption in the visible and near‐infra red regions. However, indoor photovoltaics (IPVs) utilize the light sources in interior spaces and particularly the ones adapted to the photopic eye response within the 400–800 nm range [9]. Dye molecules that show intense absorption and high absorptivity in this range are promising candidates for this application. Conventionally, Ru‐based sensitizers have been used for high performance DSSCs but their use has been limited due to cost and scarcity of Ru [10, 11]. Attention has therefore shifted to metal‐free photosensitizers due to their ability to reduce preparation costs, increase material availability and to offer tunable optoelectronic properties [7, 12]. The latter is crucial for the newly emerging low‐light application as the dye's bandgap (ideally 1.9–2.0 eV) [13] can be tuned to achieve highly efficient devices under these illumination conditions. The photosensitizer plays a crucial role of photon absorption, electronic injection and regeneration. Consequently, the positioning of the energy levels must match. The dye must show high absorptivity in regions where the emission peak of the LED/CFL light source is important.

Recombination limits the performance of DSSCs by causing charge loss. In a recent work, we have demonstrated that under low light conditions, recombination losses resulting from the systematic deterioration of the electron diffusion length become particularly critical [14]. This work demonstrated that suboptimal electrolyte composition and photoanode properties such as the crystallinity of both the blocking layer and the mesoporous layers can limit charge collection efficiency, particularly at low injection levels. We also reported that the conductivity and resistivity of the transparent conducting oxide (TCO), had little or no impact under low‐light irradiation and that high enough TiO_2_ sintering temperatures were necessary to dissolve series resistances induced by the substrate [15].

Wang et al. improved transport and suppressed recombination rate by reducing trap states through doping the nanocrystalline TiO_2_ films with varying Zn ratios [16]. Record photovoltages for IPV surpassing 1V at 1000 Lx were achieved using ZnO hierarchical microstructures as overlayers on top of mesoporous TiO_2_ as photoanodes in [Cu(dmp)_2_]^1+/2+^‐based devices [17]. While the use of design of experiments (DoE) has long been widely applied to optimize fabrication parameters in various types of DSSCs for outdoor applications [18, 19, 20], more recently, Liotier et al. [21] introduced a combined approach integrating machine learning (ML) with DoE to optimize electrolyte composition in both photochromic and conventional DSSCs. This strategy enabled significant improvements in both transparency and efficiency in this class of DSSCs. DoE provides a structured and statistically rigorous approach to exploring the parameter space with a minimum number of experiments and capturing the effects of interactions between variables, such as electrolyte composition or processing conditions, while ML uncovers more complex nonlinear relationships that are difficult to identify using conventional analysis methods. ML can predict performance across unexplored conditions and guide further experiments to accelerate convergence toward optimal formulations. This pioneering work paved the way to use similar strategies to optimize the photoanode architecture and the electrolyte composition in DSSCs for IPV [21].

Among the large number of organic dyes reported to date, **RK1** is a classic organic dye with a donor–π–spacer–acceptor structure. Its exemplary photovoltaic performance of over 10% under standard conditions has been reported [22]. This dye exhibits high extinction values, demonstrating a maximum absorption peak in the visible region at λ = 470 nm with an epsilon value of 26600 M^−1^.cm^−1^ which makes it appealing for IPVs. In addition, **RK1** has demonstrated exceptional stability in DSSCs containing an iodide‐based electrolyte, with a PCE that declines by *circa* 25% after 7000 h of accelerated aging under ISOS‐L2 conditions [12, 23].

In this context, **RK1** has been used to address the challenge of developing efficient DSSCs with iodide‐based electrolytes for indoor use. To maximize our chances of success, this work proposes a new photoanode architecture involving a mixture of nanoparticle sizes aimed at cutting recombination events at the TiO_2_/dye interface. Building on our previously work, we used DoE and ML algorithms to optimize the electrolyte for indoor application and establish an additive‐property interdependence correlation. Specifically, we employed and compared two ML optimization approaches, i.e. the support vector machine (SVM) and Bayesian optimization (BO). In addition, we carried out an optimization study to minimize recombination loss by adjusting the particle size and the surface area between the oxide and electrode, where most of the recombination events occur. To the best of our knowledge, no study has focused on understanding the impact of particle size in the photoactive layer or on finding an optimal iodide‐based electrolyte under indoor conditions. Our efforts aimed to maximize overall efficiency and increase the open circuit photovoltage, due to the necessity of achieving high photovoltages to power IoT devices.

## 2 Device Fabrication

Before we examine the results, this section will outline the methods and conditions employed in the manufacture of the devices under study. The photoanodes (made from Xop glass, FTO TEC 15) were prepared by first cleaning the substrates sequentially using Hellmanex, deionized water, ethanol and isopropanol in an ultrasonic bath for 15 min, subsequently. The substrates are dried under a N_2_‐flux and cleaned with ozone for 15 min. A TiO_2_ blocking layer is deposited by spray pyrolysis at 450°C using a 14 mM solution of Titanium (IV) bis(acetylacetonate) diisopropoxide in absolute ethanol followed by sintering for 30 min further. Consecutive layers are screen printed using an ATMA AT‐45FA printer with either a fully transparent (18NR‐T paste) or a semitransparent layer (18NR‐AO) as photoactive‐TiO_2_. Classical mesoporous‐TiO_2_ was used as a control and with the thickness of the layers varied as follows; one layer of mesoporous + one layer of scattering layer termed (1LT + O), two layers of mesoporous + one layer of scattering layer termed (2LT + O), and finally three layers of mesoporous + one layer of scattering (3LT + O). The modified photoanodes, termed the V‐configuration, were screen printed using an 18NR‐AO paste with nanoparticle size distribution ranging from 20 to 200 nm as the photoactive layer and the thickness was varied as previously explained: one, two, and three layers followed by a scattering layer labeled as 1LS + O, 2LS + O, and 3LS + O, respectively.

**FIGURE 1:**
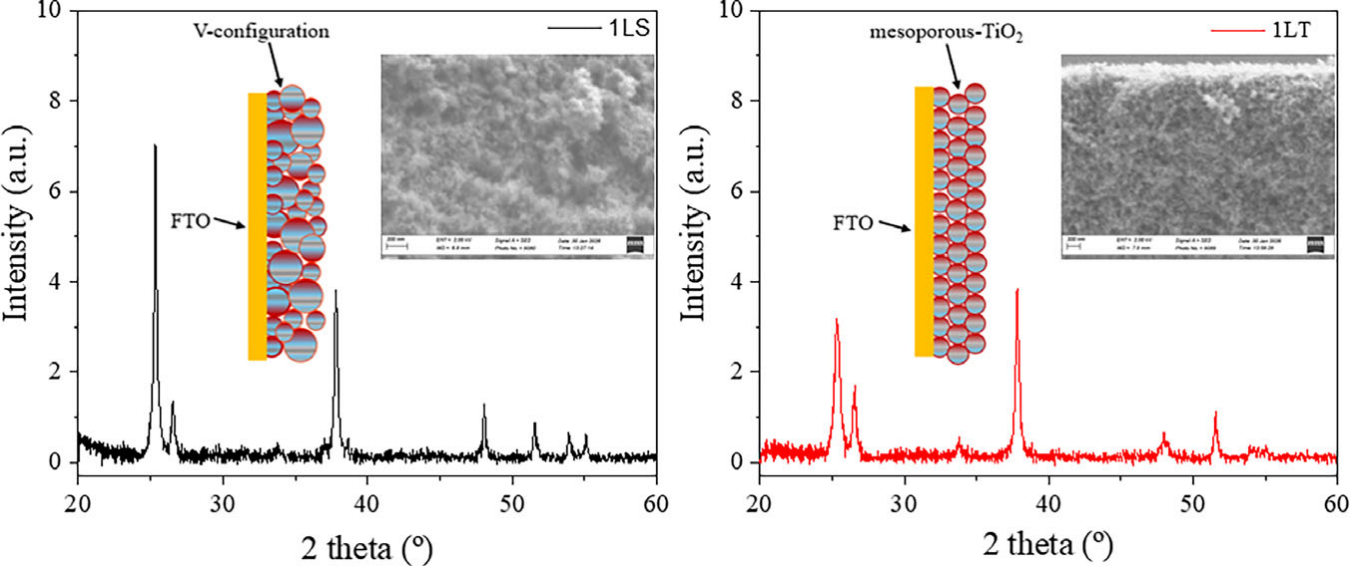
The X‐ray diffraction spectra, and inset their respective SEM images and diagramatic representations on the left (V‐configuration photoanode) with one semiopaque layer and on the right (mesoporous) one transparent TiO_2_ layer.

As shown in Figure 1 below, the 1LS and 1LT substrates show differences both in the diffraction peaks position and intensity. For example, the 25.25° 2*θ* peak is sharper for the V‐configuration electrode, suggesting larger crystallite sizes and improved crystallinity compared to its counterpart. The resulting films were sintered at 550°C for 30 min and had a thickness varying from 4 to 10 µm and an area of 0.16 cm^2^. The electrodes were then cooled down to 80°C and immersed in a dyeing bath solution containing 0.5 mM **RK1** with the ratio of chenodeoxycholic acid (CDCA) to the dye varying between 0:1, 1:1, 2.5:1, 5:1, 7.5:1, and 10:1.

The counter electrodes (made of Xop glass, FTO TEC 8) were prepared by drilling an electrolyte injection hole first, then cleaning them using a similar procedure to that applied for the photoanodes. The platinum catalyst was deposited by doctor blading a *Platisol T* (*Solaronix*) precursor solution after which the electrodes were activated at 400°C for 5 min. The photoanode and the counter electrode were assembled in a sandwich configuration using a 60 µm‐thick Surlyn. An electrolyte solution, containing 0.03M I_2_, 0.1M LiI, 1 M BMII, 1 M tBP, and 0.1M GuSCN in acetonitrile, was injected using a vacuum pump as previously reported [14, 15]. A complete device was obtained by sealing the holes with Surlyn and a glass cover followed by creation of contacts at the cell edges. The photovoltaic parameters of the complete devices were measured using a Lightbox toolbox (Lightricity) at 500 and 1000 Lx. This equipment provides 5000 K CRI70 illumination with a spectral coincidence of 0.91 with respect to the international IEC standard (IEC TS 62 607‐7‐2:2023) [24]. The certified irradiance of the Lightbox is 0.0605 and 0.3026 mW/cm^2^ at 200 and 1000 Lx, respectively. The interpolated irradiance at 500 Lx is 0.1513 mW/cm^2^. The total uncertainty of the calibration process is below <4%.

Electrochemical impedance spectroscopy (EIS) measurements were performed using a White LED with a color temperature of 6000 K which supplied a constant current while the distance between the LED and the device was varied to attain an equivalent open circuit potential at the given light intensity. The EIS measurements were carried out in the 10^5^–10^−1^ Hz frequency range with a 10 mV amplitude perturbation applied. The resulting spectra were fitted using Z‐view software (Scribner Associates, Inc.) with the transmission line model containing an extended element (DX1) as the equivalent circuit [25].

## 3 Results and Discussion

### 3.1 Classical Photoanode Optimization

The **RK1**‐based DSSCs were first incorporated with an electrolyte previously reported in **N719**‐based devices. This electrolyte contained 0.03 M iodine (I_2_), 0.1 M lithium iodide (LiI), 1 M 1‐butyl‐3‐methylimidazolium iodide (BMII), 0.1 M guanidinium thiocyanate (GuSCN), and 1 M 4‐*tert*‐butylpyridine (*t*BP), and was intended for use in low‐light applications [15]. The photoanodes consisted of a 7–8 µm mesoporous layer and a 3–4 µm semiscattering layer. **RK1** contains a long C8 alkyl chain in its core which is sought to protect the TiO_2_ from the triiodide potentially acting as electron scavenger. Therefore, to start this study, we applied **RK1** in a DSSC configuration without a CDCA coadsorbent and compared to previously optimized **N719**: 1 CDCA devices.

Surprisingly, the PV performances of the two systems was comparable at 500 Lx, with the PV parameters of **N719** devices being *J*_sc_ = 45.4 µA.cm^−2^, *V*_oc_ = 557 mV, FF = 79.7%, and ŋ=13.6% and the PV parameters of **RK1** devices being 46.5 µA.cm^−2^, 549 mV, 79.9%, and 13.8%, respectively (see Figure S1). Higher iodide concentration is associated with quantitative dye regeneration whereas lower concentrations result in lower recombination and improved electronic lifetimes [26, 27]. When the concentrations of the electrolyte components were halved except for *t*BP and GuSCN which were maintained at 1  and 0.1 M, respectively, the following effect was observed: for the **N719** devices, the measured parameters were *J*_sc_ = 45.7 µA.cm^−2^, *V*_oc_ = 583 mV, FF = 79.4%, and ŋ=14.4% whereas for the **RK1** devices the parameters were *J*_sc_ = 33.6 µA.cm^−2^, *V*_oc_ = 573 mV, FF = 81.3%, and an ŋ=10.6% (see Figure S2). The reduction of I_2_, LiI, and BMII which contribute to the redox species and hence regeneration kinetics has a detrimental impact on the purely organic dye leading to reduced efficiencies. It appears that altering the electrolyte composition has a significant effect, which varies depending on the type of sensitizer, highlighting the need for specific optimization. Given the relatively low voltage open circuit (*V*_OC_) obtained under these conditions, it is hypothesized that the hydrophobic tails in **RK1** are insufficient for a proper passivation of the electrode from triiodide. Therefore, the CDCA concentration needs to be optimized for optimal performance.

CDCA has been used as an effective organic molecule for coadsorption and acts as an additive [28]. Based on the above results, the CDCA content was optimized using similar photoanodes with the dye:CDCA ratio varied as follows 0, 1, 2.5, 5, and 7.5 (see Figure S3). The optimal coadsorbent was found to be **RK1**:2.5CDCA with registered PV parameters at 500 Lx of *J*_sc_ = 48.3 µA.cm^−2^, *V*_oc_ = 571 mV, FF = 80.5%, and a PCE = 15.1%. This improved performance is due to an increased current density resulting from spacing the dye molecules on the TiO_2_ surface, limiting aggregation and quenching of the excited states [29].

The thickness of the TiO_2_ films has been investigated for DSSCs under standard conditions. Thicker photoanode substrates have been reported to be ideal for high performance devices [30]. In this case, the variation in thickness was achieved by screen printing either one, two, or three layers of the photoactive mesoporous layer resulting in substrates that were 3–4 , 7–8,  or 10–11 µm‐thick, respectively. These photoanodes were used with an optimized dye bath composition to fabricate DSSC that were measured at 500 Lx.

The current–voltage characteristics of thinner films, i.e. 1LT + O resulted in slight improvements, *J*_sc_ increased to 50.6 µA.cm^−2^, *V*_oc_ increased to 585 mV, and FF increased to 80.8% leading to an efficiency of 16.2%. An increased TiO_2_ film, i.e. 3LT + O resulted in a *J*_sc_ of 52.1 µA.cm^−2^, *V*_oc_ of 562 mV, a FF of 81.1%, and hence a PCE of 16.1% (see Figure S4). This result shows that increasing the film thickness leads only to a small increase in the current due to more dye molecules attached onto the surface but also a loss in the photovoltage, which is a crucial parameter for indoor applications. This finding highlights the importance of electron recombination losses when the thickness exceeds the diffusion length [31, 32]. Moreover, a thinner substrate is also a more viable economic option for industrial‐scale manufacturing.

### 3.2 PV Performance of V‐Configuration Photoanodes

Large grain particles have been used in perovskite solar cells technology to enhance the transport and optical properties of the MAPbI_3_ films leading to improved PV performance while preserving their crystallinity [33]. With this in mind, a commercial paste with a combination of large and small nanoparticles was used instead of the mesoporous layer for the first time (**V‐configuration**). The classical photoanode bearing particles in the 20 nm range was used as a control against the newly proposed architectural design. A fully opaque light scattering layer was maintained to enhance optical absorption.

**FIGURE 2:**
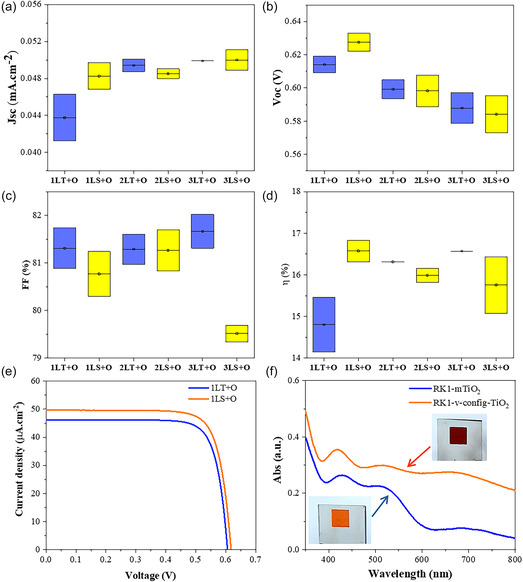
PV parameters (a) J_sc_, (b) *V*_oc_, (c) FF, and (d) PCE comparison between classical architecture with mesoporous TiO_2_ (LT + O) versus the new architecture (V‐configuration) LS + O at 500 Lx. The electrode thickness was increased by increasing the number of screen‐printed layers 1,2, and 3, respectively. (e) J(V) comparison between classical m‐TiO_2_ versus V‐configuration based devices at 500 Lx under similar experimental conditions and (f) their respective UV–Vis spectra of sensitized photoanodes.

As shown in Figure 2 below, apart from the classical single‐layered mesoporous photoanodes, the current density of all the architectures remains comparable regardless of variations in electrode thickness. The V‐configuration photoanodes demonstrate significantly higher *V*_oc_ values at 500 Lx than their single‐layered mesoporous counterparts. As the thickness increases, the photovoltage diminishes progressively. Despite the relatively high short‐circuit photocurrents of 1LS + O devices being accompanied by high photovoltage, the fill factor remains unaffected suggesting a good interparticle connection and a high‐quality film. Indeed, the best device which used a nonoptimized electrolyte achieved a *J*_sc_ = 49.7 µA.cm^−2^, *V*_oc_ = 626 mV, and FF = 80.9% giving a PCE of 16.95% at 500 Lx. This higher photocurrent is also linked to the light scattering effect of larger grains in the photoactive layer, which improves photon absorption (see Figure 2f).

**FIGURE 3:**
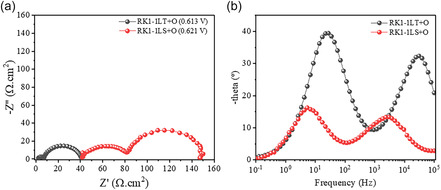
(a) Nyquists plots of **RK1**‐based devices using a classical mesoporous layer as the photoactive layer (1LT + O) versus V‐configuration architecture (1LS + O) under low irradiation conditions at open circuit conditions equivalent for indoor operation and (b) the corresponding Bode plots.

To understand the notably higher *V*_oc_ and the impact of grain sizes in V‐configuration devices compared to conventional ones, an EIS study was conducted and a comparison was drawn. Figure 3 shows illustrative impedance spectra for the two configurations at approximately the same value of the voltage.

Typical impedance response in DSSCs show three characteristic arcs on the real axis of the frequency domain. The high frequency relates to the cathodic reactions, the mid‐frequency arc to the transport and recombination resistance at the TiO_2_/dye/electrolyte interface and the low frequency one to the migration of redox species in the electrolyte [34, 35]. In this case, the low‐frequency arc is not observed in both architectures suggesting an efficient regeneration unlimited by the movement of charged species in the electrolyte [36, 37] under a low‐light regime. The new architecture has a tendency to show enhanced series resistance values. This observation could be detrimental for outdoor operation but this is not problematic under indoor lighting [15]. The high‐frequency arc appears larger than in a classical mesoporous TiO_2_‐based devices. This indicates that the arc is responsible not only for the cathodic reaction but also for the interaction and charge transfer at the TiO_2_/FTO interface. Indeed, the mixed particle sizes favors the formation of pin holes resulting in an increased charge transfer resistance at this interface. The lower recombination rate can be explained by the reduced surface area of the V‐configuration, which contains larger particles, as well as the enhanced crystallinity (see Figure 1). The Bode plot on the other hand gives information on effective electronic lifetime $\left(\tau_{e}=\frac{1}{2\pi f}\right)$ denoting an extended lifetime in V‐configuration photoanodes at approximately the same value of the voltage hence corroborating decreased recombination events.

In order to extract physical meaning from the operational processes in DSSCs, the EIS data is often fitted to an equivalent circuit by simplifying kinetics. In this case, the data was fitted to the transmission line model proposed by Bisquert and coworkers but unfortunately this was not possible due to a lack of a vivid transport line in the mid‐frequency arc [25]. Therefore, Nyquists plots were fitted with circles to estimate the resistive and capacitive behavior which was found to correspond to true chemical capacitance that varies exponentially with the applied potential [34, 38] (see Figure S5).

### 3.3 Optimization of IPVs by Machine Learning

Throughout this process, we have been working with an electrolyte containing five components in a mixture. Optimizing one component versus one factor at a time can be time‐consuming and resource‐intensive process. Here, we improve on predictions and uncertainties, i.e. exploitation and exploration upon a strategy that we previously reported by applying it here to a more complex system [21]. We also implemented a BO strategy to benchmark its predictive performance against SVM ML model. V‐configuration electrodes were used to train the model applying a DoE approach to gain an overview of several factor and variables simultaneously.

As a reminder, the iodine‐based electrolyte so far contains the following compounds in acetonitrile solvent: 0.03 M I_2_, 0.1 M LiI, 1 M BMII, 1 M *t*BP, and 0.1 M GuSCN. The concentrations of the components were varied as follows: iodine 0.01, 0.025, and 0.04 M, LiI (0, 0.1 and 0.2) M, BMII (0.5, 1.0, 1.5) M, *t*BP (0.5, 1.0, 1.5) M, and GuSCN (0, 0.1, 0.2) M. With five parameters and three levels each, an exhaustive exploration of the parameter space would require 243 experiments (3^5^ combinations). However, an educated guess through DoE identified 25 experiments of interest as shown in Table S1. At least two devices were fabricated for each electrolyte configuration under similar experimental conditions discarding leaking devices to minimize errors and characterized at 500 Lx.

As shown in Table S2 (or Figure S6), the measured PV parameters indicate a *J*_sc_ ranging between 33–66 µA.cm^−2^. The lowest current densities were obtained using an electrolyte (experiment 6, see Table S1) containing 0.025 M I_2_ and 0 M LiI. On the other hand, this electrolyte produced higher *V*_oc_ values of around 674 mV due to a higher *t*BP concentration (1.5 M) which is known to have an upward bandshift effect [5,39]. The highest *J*_sc_ values were achieved with an electrolyte containing 0.01 M I_2_, 0.1 M LiI, 1.5 M BMII, and 0.5 M *t*BP (see experiment 8, Table S1). This composition resulted in a current density of 66.7 µA.cm^−2^, a *V*_oc_ = 623 mV, a FF = 81.7%, and a PCE = 22.45% at 500 Lx. This result ranks among the best reported in the field for liquid iodine‐based electrolytes. Most record efficiencies for this type of system fall within the 18%–29% range at light intensities between 500 and 1000 Lx [40, 41], with values typically exceeding 30% only at higher illuminance levels (approximately 1500 Lx). However, it should be noted that direct comparison remains challenging as these performances are reported under different conditions, including variations in light sources (e.g. LEDs and/or fluorescent lamps) [42, 41, 43].

**FIGURE 4:**
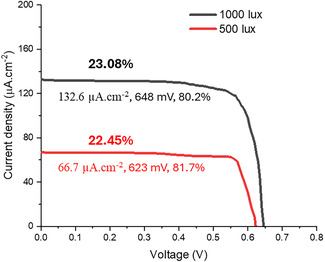
The current–voltage characteristics of the best electrolyte composition identified in the experimental plan showing the response at 500 and 1000 Lx light intensities.

When measured at a higher light intensity of 1000 Lx, these devices achieved a *J*_sc_ of 132.62 µA.cm^−2^, a *V*_oc_ = 648 mV, and a FF = 80.2%, hence a PCE = 23.08% as shown in Figure 4 below. This result suggests that reducing the iodine concentration is necessary for high performance iodine‐based IPVs by minimizing parasitic photon absorption. The electrolyte composition certainly demonstrates high visual transparency enhancing dye absorption.

The recombination resistances (*R*_*rec*_) of the fitted‐circles in the impedance spectra were determined to deduce relationship between properties and components. The impedance measurements to train the models were performed at a fixed voltage (610 mV) in order to make comparisons. We first use the correlation between components and measured parameters using a Pearson coefficient chart (see Figure S7). As expected, the current and the current density shows a trivial correlation of 1. Several negative correlations are also observed indicating inversely proportional relationships. According to the chart, the strong effect of LiI and *t*BP on the *J*_sc_ is consistent with the design principles of DSSCs [44]. The FF of the devices seems to be mostly influenced by the amount of *t*BP showing a good linear correlation. Recombination resistance (*R*_*rec*_) is strongly related to the *V*_oc_ [45]. To further quantify the influence of each parameter, an analysis of variance (ANOVA) was performed on the dataset. The results indicate that LiI, BMII, and *t*BP are statistically significant (*p* < 0.05) in determining the photovoltage of the devices. Conversely, I_2_ and GuSCN have almost no influence on the final PV parameters with a *p*‐value > 0.05 (see Figure S8). Another statistical parameter used to evaluate ANOVA is the F‐value that tends to separate the total variability in the data into two components. The first is between‐group variance which may result from variations in experimental conditions such as different compositions, treatments, or other process parameters. Second, there is within‐group variance also referred to as natural spread or noise within each group. The F‐value is a ratio of these two components. If the between‐group variability is much larger than the within‐group variability, this suggests that at least one group mean is significantly different (see Figure S8). This indicates that the differences between groups are statistically significant rather than arising from random noise.

Although the gradient boosting regression model was able to predict regions of interest in the parameter space, visualizing the influence of the five electrolyte components simultaneously remained challenging. Representing a five‐dimensional parameter space in a clear and intuitive way is not straightforward. To address this, we first optimized an XGBoost regression model and subsequently used it to generate predictions across the entire parameter space. These predictions were then visualized as three‐dimensional surfaces using the Plotly library, allowing us to explore the relationships between selected component combinations while keeping the other parameters fixed. Among the different projections tested, the representation involving GuSCN, BMII, and LiI provided the clearest visualization of the predicted performance landscape. As shown below, the model suggests that optimal photovoltaic parameters are obtained at relatively low concentrations of these three components.

**FIGURE 5:**
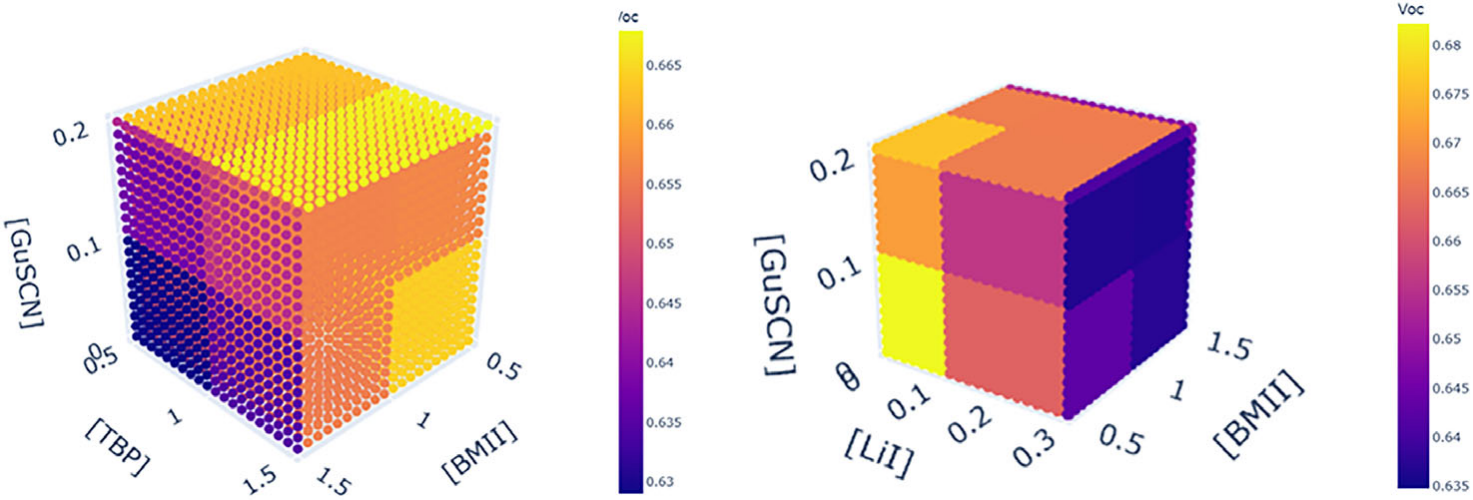
Three‐dimensional mapping of predicted photovoltaic performance generated by the optimized XGBoost gradient boosting regression model trained on the DoE experimental dataset.

The optimized XGBoost model used the following parameters: learning_rate = 0.01, max_depth = 4, n_estimators = 200, and subsample = 0.8. The model achieved an R^2^ score of 0.64 with a root mean square error (RMSE) of 0.0124 and a mean absolute error (MAE) of 0.0098. Although the predictive accuracy remains moderate, the model provides useful guidance for identifying promising regions of the parameter space and directing subsequent experimental optimization (see Figure 5).

BO, typically using a Gaussian process as a surrogate model, combines an objective function with an acquisition function to guide experiments or simulations. This allows efficient exploration of the parameter space and identifies the most promising conditions to achieve optimal outcomes in costly or time‐consuming processes. Using the data obtained from the DoE, the BO model was trained as shown in Figures S9–S11, which show the measured and predicted pair plots for each PV parameter. The ad hoc ML optimizations were carried out targeting the *J*_sc_, *V*_oc_, and PCE separately (see Table S3). A set of five predicted experiments and predicted outcomes was deduced and tested in devices. To optimize the PCEs, the model predicted a conversion efficiency of 23.5% at 500 Lx using an electrolyte containing I_2_ = 0.05 M, LiI = 0.23 M, BMII = 0.63 M, *t*B*P* = 1.09 M, and GuSCN = 0.21 M giving the following PV parameters, *J*_sc_ = 62.7 µA.cm^−2^, *V*_oc_ = 595 mV, FF = 77.9, and PCE = 19.22%. On the other hand, an improved current density (85.6 µA.cm^−2^) was predicted using an electrolyte containing 0.05 M I_2_, 0.21 M LiI, 1.5 M BMII, 1.47 M *t*BP, and 0.35 M GuSCN. The devices registered the following current–voltage characteristics: *J*_sc_ = 60.11 µA.cm^−2^, *V*_oc_ = 596 mV, FF = 77.4%, and hence PCE = 18.32%.

Finally, the SVM model was applied alongside with the Bayesian model to help improve the photovoltage of devices which is crucial parameter for powering IoTs. Using an electrolyte with 0 M I_2_, 0 M LiI, 0.26 M BMII, 1.61 M *t*BP, and 0.5 M GuSCN, a high photovoltage of 830 mV was projected. According to the operational principle, this electrolyte is unfavorable due to a lack of redox species. However, surprisingly, this formulation gave a *J*_sc_ = 48.4 µA.cm^−2^, *V*_oc_ = 683 mV, FF = 74.05%, and PCE = 16.19% at 500 Lx. At 1000 Lx, the device attained 97.3 µA.cm^−2^, *V*_oc_ = 713 mV, FF = 72.23%, and PCE = 16.55%. Although this result is surprising, it can be explained by the fact that BMII can provide iodide ions that is part of the counter‐anion of the salt. In solution, BMII dissociates into BMIm^+^ and I^−^, resulting in the presence of free iodide ions without any chemical decomposition. Following the work of Kong et al., which demonstrates the formation of I^−^/I_3_^−^ from the decomposition of 1‐methyl‐3‐propylimidazolium iodide (MPII) [46], we hypothesize that a small amount of I_3_^−^ may form through oxidation of I^−^ (by oxygen or electrochemical reactions), but the concentration is in that case too low to obtain a high‐performance DSSC electrolyte. We found that I_2_‐free electrolytes (see Experiment 1 of Table S3c), quickly after their preparation, exhibit an absorption peak at around 360 nm in the UV–Vis spectra, characteristic of the presence of I_3_^−^ species. This indicates its in‐situ formation within the electrolyte solution via decomposition of BMII (see Figure S12 in the ESI). This observation is consistent with recent results published during the review process of this article [47]. Eliminating BMII component in the best electrolyte formulation in this study, electrolyte 8 (Table S1), resulted in a composition containing 0.01 M I_2_, 0.1 M LiI, and 0.5 M *t*BP. At 500 Lx, this device gave *J*_sc_ = 63.3 µA.cm^−2^, *V*_oc_ = 591 mV, FF = 73.2%, and PCE = 18.11% and 127.3 µA.cm^−2^, 616 mV, 73.0%, and 18.90% at 1000 Lx, respectively (see Figure S13). The *V*_oc_ and the FF suffered most, thus depicting the crucial role of BMII in an electrolyte formulation as a TiO_2_ surface passivator and in creating a surface dipole moment [48].

Although neither model applied was able to improve the *J*_sc_ and PCE values in the devices, the photovoltages attained from ML predictions were the highest achieved in this study using **RK1** dye. The electrolytes developed in this work reveal that low iodide concentrations allow for improved *V*_oc_ and relatively high performance. This study opens a new insight on the fundamental operating principles, suggesting the formation of the I^−^/I_3_^−^ couple at levels sufficient to achieve decent performance in devices under low illumination, with BMII becoming the main contributor to the redox species.

## 4 Conclusion

In summary, DSSCs show promises for powering IoTs but much work is needed to understand how they operate under ambient lighting conditions. Although design principles for outdoor operation have been clearly established, they cannot be extrapolated to indoor environments due to changes in light source emissions and physics at low light intensities. In order to optimize the performance of organic dyes such as **RK1**, which have not yet been reported to operate in indoor spaces, it is essential to optimize both the photoanode and the electrolyte in order to achieve high‐efficiency devices. When this work began, energy conversion efficiencies obtained with **RK1** were below 14%, with an average photovoltage of 550 mV at 500 Lx. However, after optimizing the photoanode and using ML optimization approaches to tune the electrolyte, efficiencies increased to 23%, with a *V*_oc_ exceeding 680 mV at this light level. Furthermore, we discovered that a higher *V*_oc_ (713 mV) can be attained at a higher light level of 1000 Lx when the redox mediator is formed in situ by decomposition of BMII. We also highlight that the V‐configuration for the photoanode obtained by mixing nanoparticle with size distribution ranging from 20 to 200 nm proved to be an efficient strategy for reducing recombination under low‐light conditions, hence improving performance. Future work will focus on understanding the tradeoff between transport and recombination in this novel architecture. We believe that the two ML approaches presented here can be extended to other classes of dyes, electrolyte systems and other photovoltaic technologies. In particular, SVM model was successfully applied previously to photochromic dyes, a class of dyes markedly different from **RK1**, as well as to TEMPO‐based electrolytes, which is a purely organic redox system. While this approach is generalizable for other systems, a systematic model comparison could further improve performance and avoid overfitting or underfitting. However, it still requires at least 50 data points for reliable results that lead to better prediction performance.

## Funding

This work was supported by Agence Nationale de la Recherche (Grant ANR‐24‐MER3‐0001), H2020 European Research Council (Grant 832606), Ministerio de Ciencia e Innovación of Spain (Grant PID2022‐140061OB‐I00), Fundación para la promoción de la investigación y la tecnología (Grant PREP2022‐000273), and European Regional Development Fund (Grant PCI2024‐153456).

## Conflicts of Interest

The authors declare no conflicts of interest.

## Supporting information

Supplementary Material

## Data Availability

The data are available in ESI and from the authors upon request. The code for Bayesian optimization and data are available on GitHub: https://github.com/RivasAntonio/ML‐Assisted‐Iodide‐based‐Electrolyte‐Optimisation‐and‐Photoanode‐Architecture‐for‐DSSC. https://github.com/caumaitre/opti_Indoor.
