# Supplementary material for: Machine Learning‐Assisted Optimization of Iodide Electrolytes for Efficient Indoor Dye‐Sensitized Solar Cells with Engineered Photoanodes
Source: Small Sci. 2026 Jun 13;6(6):e70324. doi: 10.1002/smsc.70324 (PMC13274309; doi:10.1002/smsc.70324)
Supplement: Supplementary file 1 — Supplementary Material [file SMSC-6-e70324-s001.pdf]

Machine Learning-Assisted Optimization of Iodide Electrolytes for Efficient Indoor Dye-Sensitized Solar Cells with Engineered Photoanodes.

Valid Mwalukuku[1,*], Antonio R. Blanco[1], Cyril Aumaître[2], Yann Kervella[2], Said Hamad[1], Renaud Demadrille[2,*], Juan A. Anta[1,*]

[1] Center for Nanoscience and Sustainable Technologies (CNATS). Department of Physical, Chemical, and Natural Systems, Universidad Pablo de Olavide, Sevilla 41013, Spain

[2] IRIG-SyMMES, Université Grenoble Alpes, CEA, CNRS, Grenoble INP, Grenoble 38000, France

E-mails: vmmwa@upo.es; renaud.demadrille@cea.fr; anta@upo.es

Materials

Glass substrates – photoanodes, FTO TEC 15 (12 - 14 Ω/□, Pilkington TEC Glass) and counter electrodes, FTO TEC 8 (6 - 9 Ω/□, Pilkington TEC Glass), Hellmanex (Hellma®Analytics), milli-Q water, absolute ethanol (VWR), 2-propanol (Panreac AppliChem), Platisol T (Solaronix), titanium diisopropoxide bis(acetylacetonate) (C16H28O6Ti) (Sigma-Aldrich - **17927-72-9**), TiO2 paste 18 NR-T (Greatcell Solar), TiO2 paste 18 NR-AO (Greatcell Solar), ruthenizer 535-bisTBA (N719) (Solaronix), RK1 dye, chenodeoxycholic acid (CDCA) (Sigma-Aldrich - **474-25-9**), iodine (I_2) (≥99.8% Sigma-Aldrich - **7553-56-2**), lithium iodide (LiI) (≥99.9% Sigma-Aldrich **10377-51-2**), 1-butyl-3- methylomidazolim iodide (BMII) (99% Sigma-Aldrich **65039-05-6**), acetonitrile (CH3CN) (≥99.5% Sigma-Aldrich - **75-05-8**), guanidine thiocyanate (GuSCN) (≥99% Sigma Aldrich **593-84-0**), 4-tert-butylpyridine (TBP) (98% Sigma-Aldrich - **3978-81-2**), meltonix 60 µm (Solaronix).

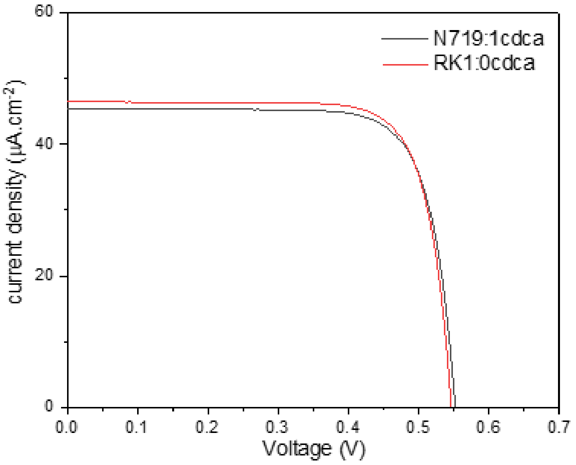

Fig. S1: I-V curves of DSSCs based on an optimised ***N719****-based device versus unoptimized* ***RK1****-based device.*

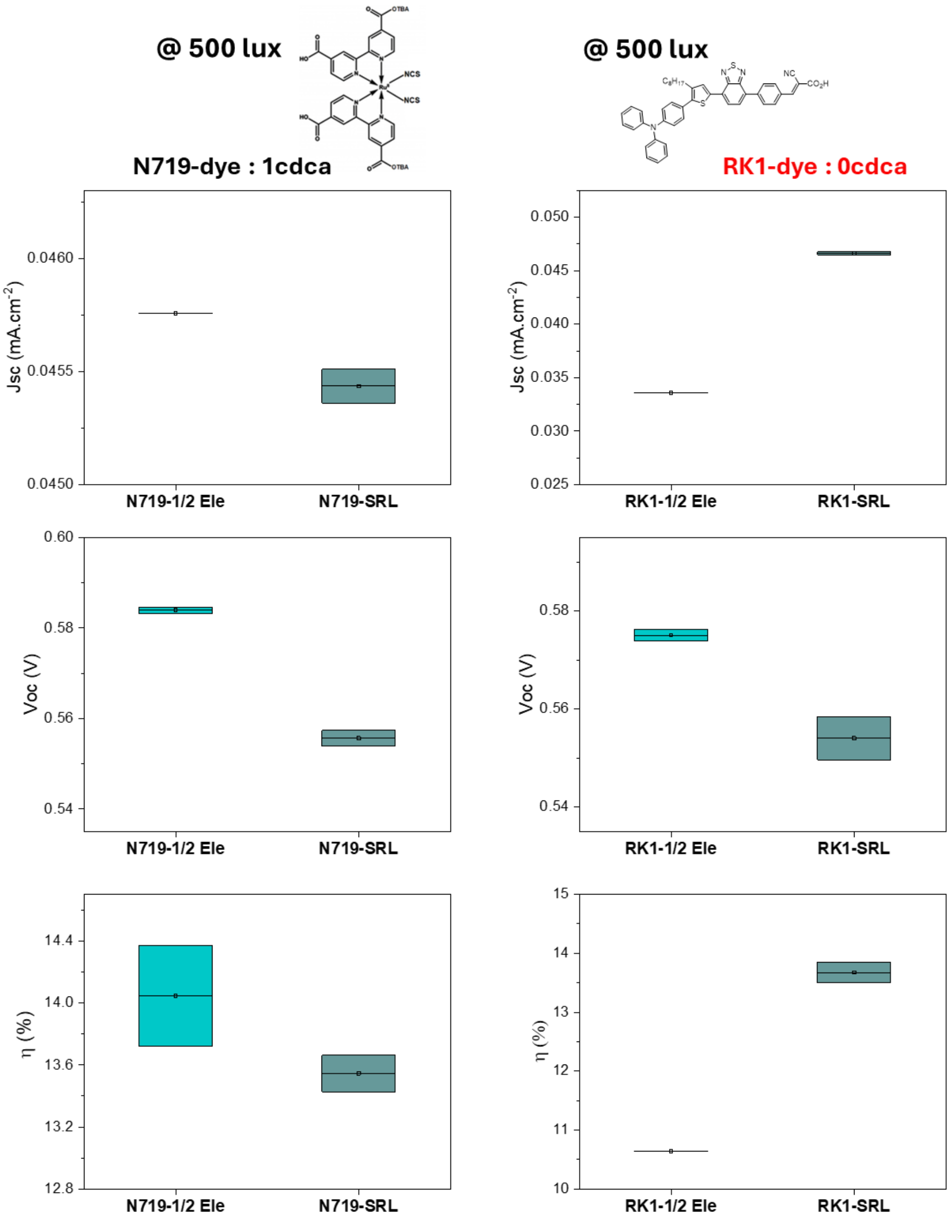

*Fig. S2: Effect of halving the electrolyte composition in **RK1**- versus **N719**-based devices. SRL is an electrolyte reported containing 0.03 M I_2, 0.1 M LiI, 1 M BMII, 0.1 M GuSCN and 1 M tBP in acetonitrile. ½ Ele means all components are halved except tBP and GuSCN that are maintained at 1 M and 0.1 M, respectively.*

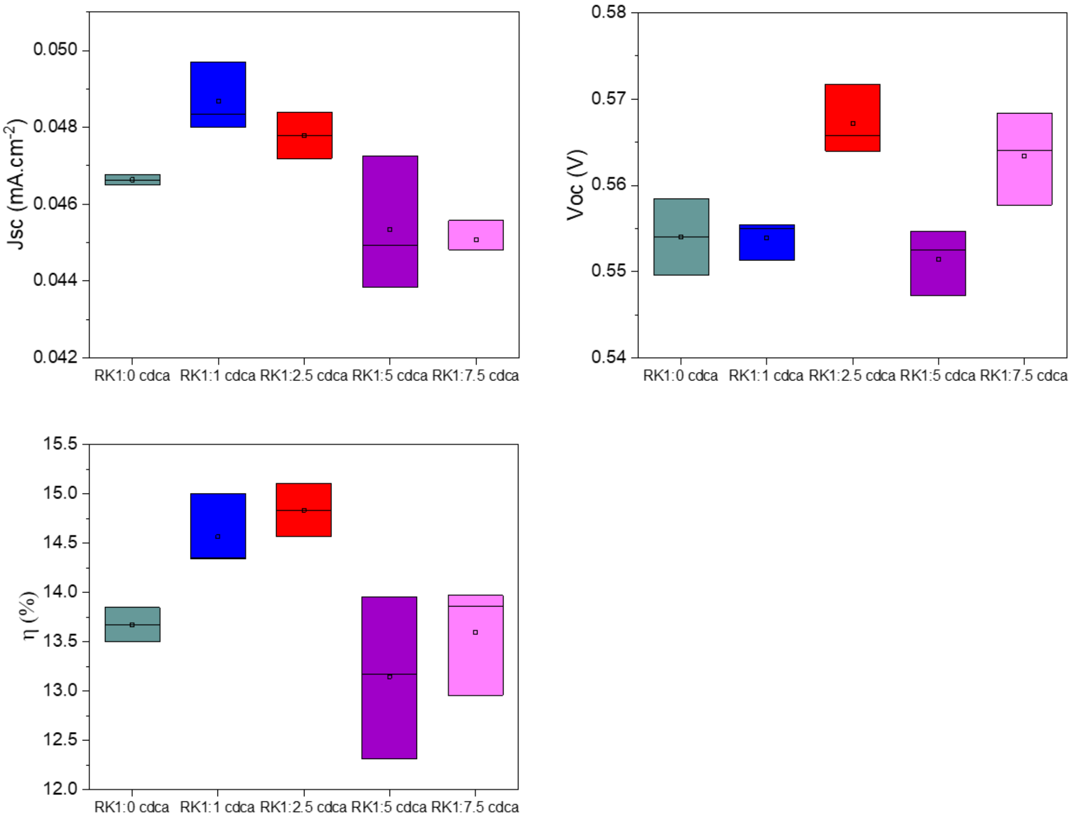

Fig. S3: Optimization of cdca concentration in ***RK1****-based devices.*

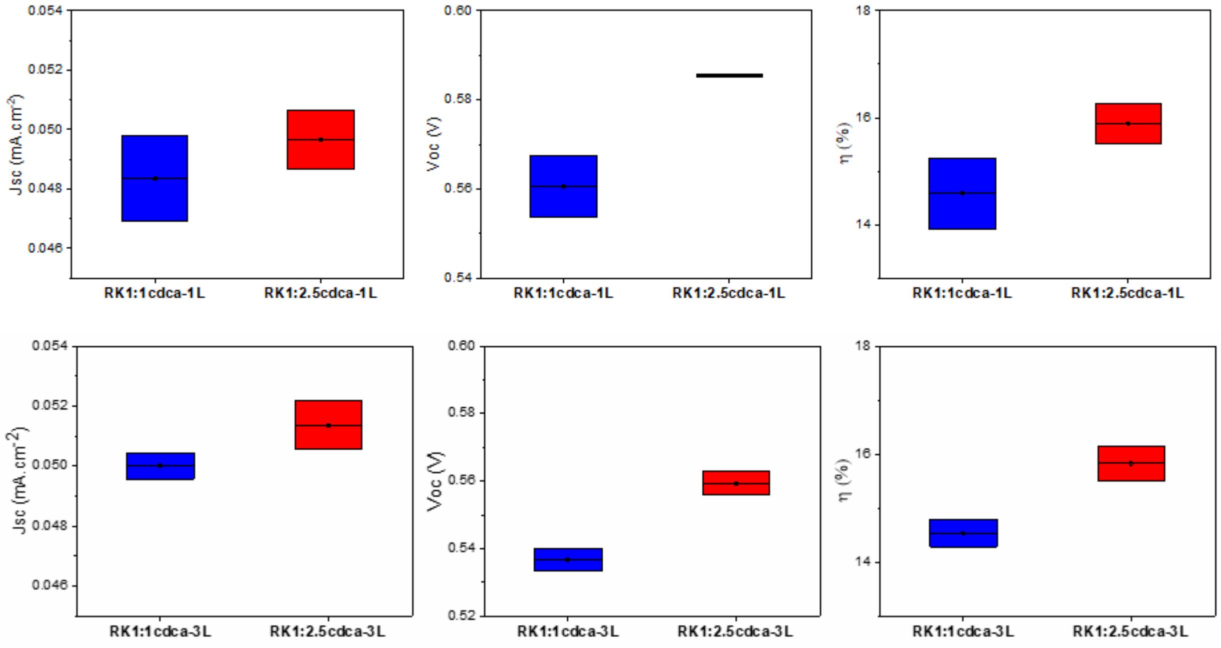

Fig. S4: Effect of varying the electrode thickness on photovoltaic parameters (above) single layers and (below) three screen-printed layers.

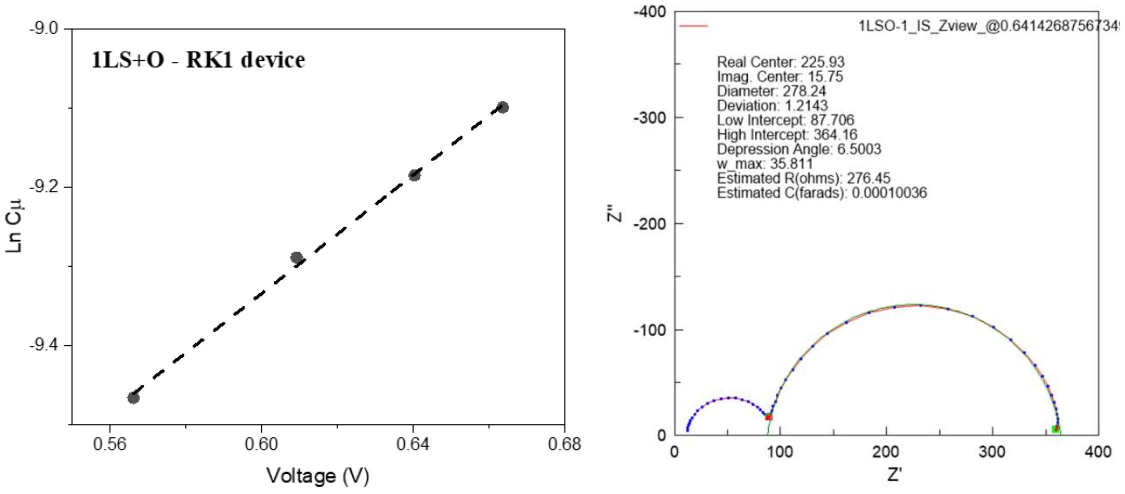

Fig. S5: (left) A plot of the chemical capacitance versus the applied voltage from fitting circles in the recombination arcs. (on the right) An example of fitted circle to an -RC- element.

Design of Experiments (DoE)

Table S1: Experimental design table for a 5-parameter and 3-level orthogonal array. Please note that the colour indicates the level and the corresponding value in the table.

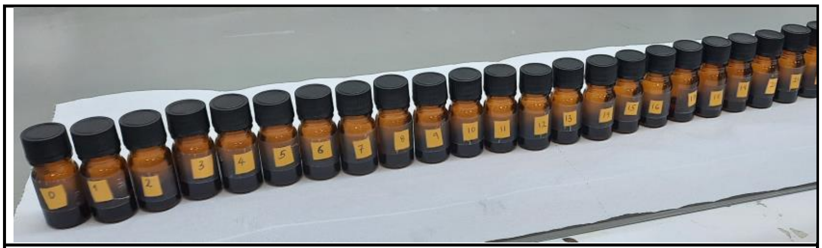

Molar concentrations					
**experiment**	**[I2]**	**[LiI]**	**[BMII]**	**[TBP]**	**[GuSCN]**
**0**	0.01	0.1	1.5	1.5	0.1
**1**	0.01	0.1	0.5	1.0	0.1
**2**	0.025	0.0	1.5	0.5	0.2
**3**	0.04	0.1	0.5	1.5	0.1
**4**	0.04	0.3	1.5	1.5	0.0
**5**	0.025	0.0	1.5	1.5	0.0
**6**	0.025	0.0	1.0	1.5	0.0
**7**	0.025	0.0	1.0	1.5	0.2
**8**	0.01	0.1	1.5	0.5	0.0
**9**	0.04	0.1	1.5	1.5	0.0
**10**	0.04	0.0	1.5	0.5	0.2
**11**	0.04	0.0	0.5	0.5	0.1
**12**	0.04	0.3	0.5	0.5	0.1
**13**	0.025	0.3	0.5	0.5	0.0
**14**	0.025	0.0	0.5	1.5	0.0
**15**	0.01	0.1	1.0	0.5	0.2
**16**	0.025	0.1	1.0	1.0	0.2
**17**	0.01	0.1	1.0	1.0	0.2
**18**	0.04	0.0	1.0	1.5	0.2

**Level 1**
**Level 2**
**Level 3**

**19**	0.01	0.1	1.5	0.5	0.2
**20**	0.04	0.1	0.5	1.5	0.2
**21**	0.025	0.3	0.5	0.5	0.0
**22**	0.01	0.1	0.5	0.5	0.0
**23**	0.04	0.1	1.0	1.0	0.0
**24**	0.01	0.3	1.5	0.5	0.2

Table S1 above details the Design of Experiment approach and multidimension visualization
Experimental design and dataset generation

The experimental dataset was generated using an orthogonal Taguchi L25 fractional factorial design to efficiently sample the five-dimensional electrolyte composition space while minimizing the number of experimental runs.
The five investigated input variables were: iodine concentration [I_2], lithium iodide concentration [LiI], 1-butyl-3-methylimidazolium iodide concentration [BMII], tert-butylpyridine concentration [TBP] and guanidinium thiocyanate concentration [GuSCN]. Each parameter was explored at three levels selected based on chemical compatibility constraints and prior experimental knowledge of DSSC electrolyte formulations as shown in Table S1 above. Each composition was fabricated in duplicate to assess reproducibility, yielding approximately 50 experimental data points used for machine learning model training. No blocking was applied, and experiments were conducted in randomized order to minimize systematic bias.

Support Vector Machine (SVM)
Data preprocessing and generation of 3D maps of targeted response.

Prior to model training, all input features were normalized using standard scaling, the dataset were randomly separated in a training and a test set for training and scoring of the model. the relationship between electrolyte composition and device performance was modeled using an Extreme Gradient Boosting (XGBoost) regressor.

The trained model approximates the nonlinear response function:

$$y = f([I_2], [LiI], [BMII], [TBP], [GuSCN])$$

where:

y is the predicted photovoltaic metric (Voc / Jsc / FF / PCE depending on the target) and f is the nonlinear surrogate model learned by the XGBoost algorithm.

Hyperparameter optimization was performed using GridSearchCV to identify the best-performing model configuration.
Unlike conventional response surface methodology, no explicit analytical polynomial model was fitted.
To visualize the high-dimensional response surface, three-dimensional projections were generated for all combinations of three input variables.

For each 3D projection:

- Three parameters were varied uniformly across their experimental ranges using 20 equally spaced values per dimension.
- The remaining two parameters were fixed at their average values across the experimental dataset.

This corresponds to evaluating slices of the five-dimensional response function:

$$y = f(x_i, x_j, x_k, \bar{x}_l, \bar{x}_m,)$$

Where $\bar{x}_l$ and $\bar{x}_m$ represent the mean values of the fixed parameters.

A regular prediction grid of 8000 (20^3) points was generated for each of the 10 projections. Predictions were computed at each grid point using the trained XGBoost model.
The predicted response values were visualized as interactive three-dimensional scatter maps using the Python Plotly library.
Colour gradients represent predicted performance values, allowing identification of the local maxima and the regions of interest for further experimental exploration.

To ensure transparency and reproducibility, the complete Python workflow used for this publication is publicly available at:

(https://github.com/caumaitre/opti_Indoor)

Table S2: Showing PV parameters of **RK1**-based DSSCs using **V-configuration** photoanodes at 500 lux.

**Electrolyte screening Results**						
**cell**	**I_{sc} (µA)**	**J_{sc} (µA.cm^{-2})**	**V_{oc} (V)**	**FF (%)**	**PCE (%)**	R_{rec}
0 - 1	9.64	60.24	0.648	73.4	18.95	572.8
0 - 2	9.36	58.53	0.639	73.6	18.17	
1 - 1	9.50	59.40	0.659	73.7	19.03	798.5
1 - 2	9.68	60.47	0.655	73.5	19.21	
2 - 1	8.43	52.66	0.647	79.2	17.84	924.6
2 - 2	8.43	52.66	0.631	77.7	17.06	
3 - 1	9.38	58.63	0.653	77.0	19.45	1114
3 - 2	8.71	54.42	0.655	78.5	18.47	
4 - 1	10.00	62.53	0.622	75.0	19.26	4556
4 - 2	9.62	60.09	0.630	73.5	18.36	
5 - 1	5.95	37.19	0.657	80.5	12.99	756
5 - 2	6.88	42.97	0.656	79.1	14.72	
6 - 1	6.61	41.28	0.675	81.4	14.97	1345
6 - 2	5.29	33.09	0.674	80.2	11.82	
7 - 1	6.63	41.46	0.675	77.9	14.39	1194
7 - 2	6.90	43.14	0.671	80.0	15.30	
8 - 1	10.68	66.79	0.623	81.8	22.46	1753
8 - 2	10.03	62.74	0.617	84.3	21.53	
9 - 1	8.91	55.68	0.62	76.4	17.43	609.1

9 - 2	9.02	56.35	0.622	76.4	17.67	
10 - 1	Fail	Fail	Fail	Fail	Fail	2009
10 - 2	8.83	55.20	0.630	74.6	17.14	
11 - 1	8.80	55.01	0.662	76.8	18.45	1230
11 - 2	8.66	54.15	0.669	75.4	18.05	
12 - 1	9.85	61.54	0.617	73.8	18.48	316.8
12 - 2	9.57	59.79	0.616	74.5	18.10	
13 - 1	8.47	52.94	0.612	68.5	14.68	562.7
13 - 2	8.93	55.83	0.616	69.2	15.70	
14 - 1	6.54	40.87	0.719	79.5	15.42	14210
14 - 2	6.29	39.28	0.715	79.7	14.78	
15 - 1	9.67	60.42	0.660	68.1	17.92	2670
15 - 2	9.36	58.52	0.665	67.9	17.43	
16 - 1	9.52	59.49	0.671	73.0	19.25	2097
16 - 2	8.53	53.30	0.663	73.1	17.05	
17 - 1	7.77	48.55	0.668	71.0	15.20	187400
17 - 2	8.33	52.05	0.675	71.7	16.66	
18 - 1	7.78	48.61	0.663	78.2	16.63	2012
18 - 2	7.92	49.51	0.658	75.7	16.28	
19 - 1	9.94	62.13	0.655	68.2	18.31	1637
19 - 2	9.14	57.10	0.653	69.1	17.01	
20 - 1	8.93	55.83	0.676	75.0	18.71	1730
20 - 2	9.02	56.36	0.675	75.3	18.91	
21 - 1	Fail	Fail	Fail	Fail	Fail	1379
21 - 2	8.89	55.54	0.648	71.1	16.88	
22 - 1	9.06	56.63	0.648	67. 8	16.41	1710
22 - 2	9.23	57.67	0.655	67.0	16.70	
23 - 1	Fail	Fail	Fail	Fail	Fail	854.1
23 - 2	8.83	55.21	0.643	73.9	17.3	
24 - 1	9.32	58.25	0.640	66.2	16.3	1228
24 - 2	9.35	58.42	0.651	66.5	16.7	

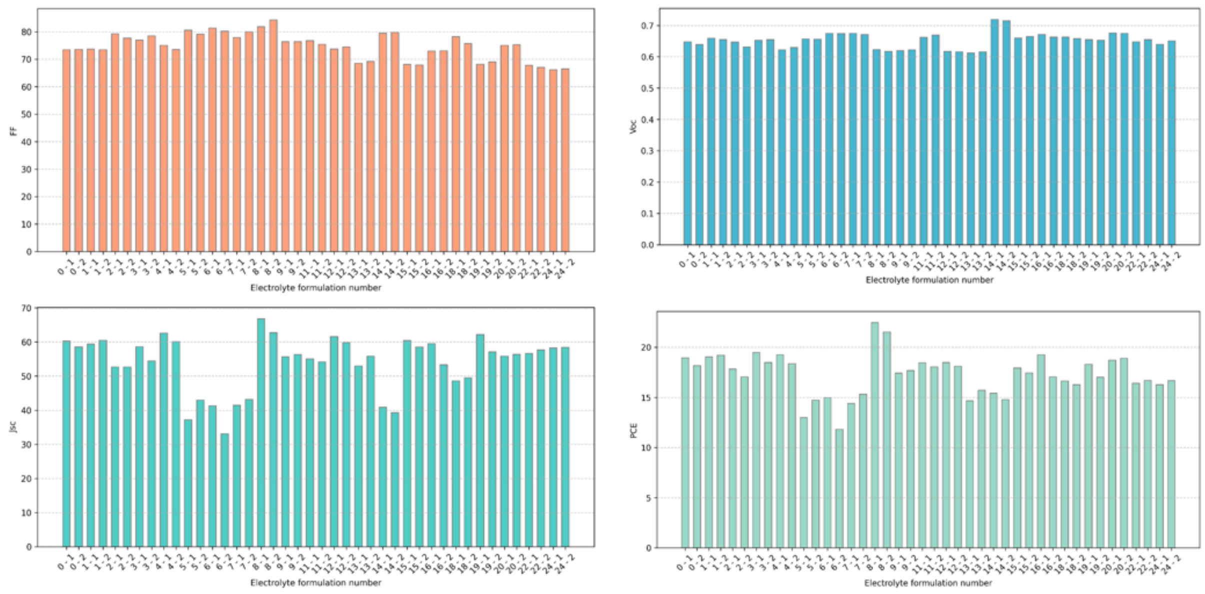

Fig. S6: Diagramatic representation of Table S2 results.

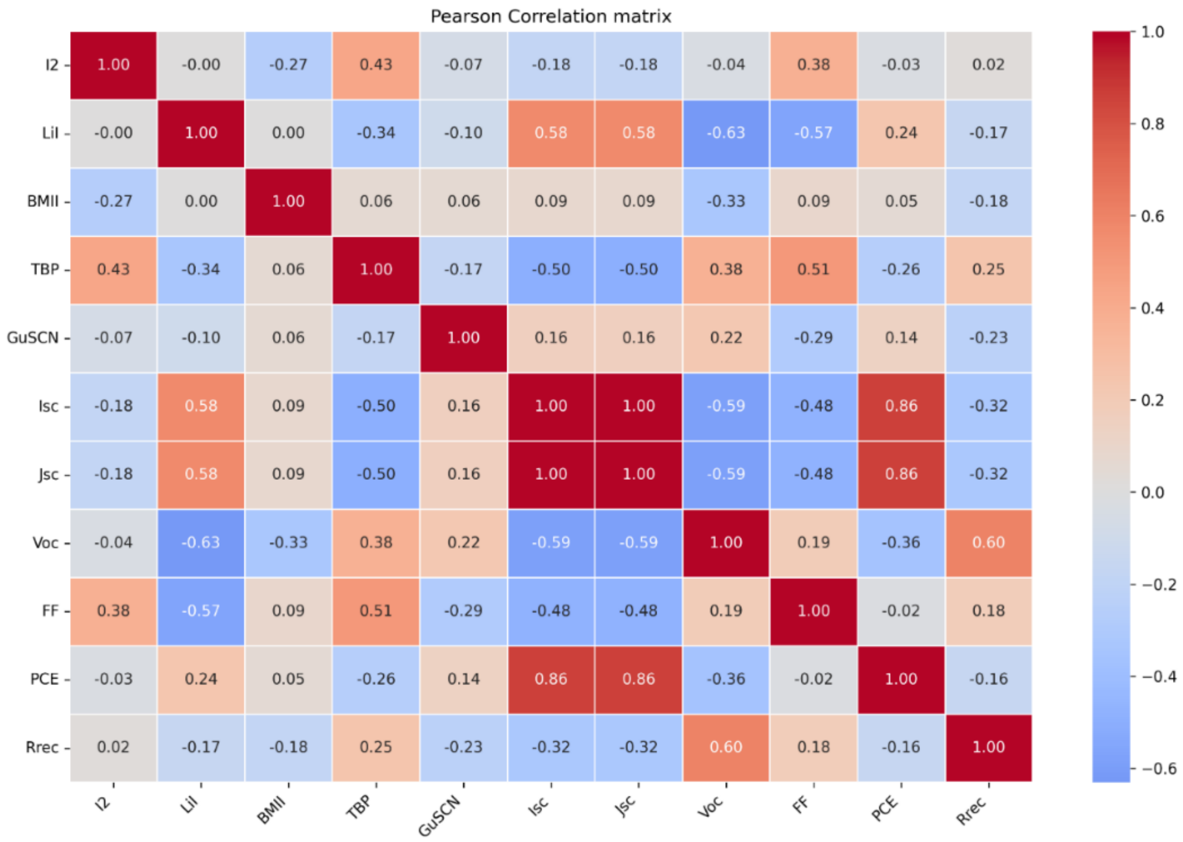

Fig S7: Showing the inter-relationship between component and PV features.

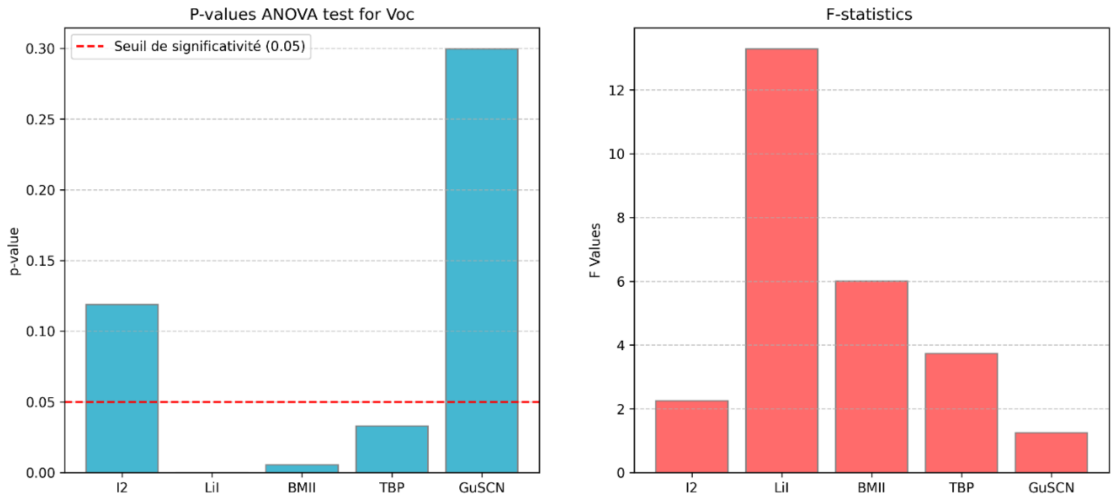

Fig. S8: ANOVA analysis.

Calculation of F-value=Variance Between Groups/Variance Within Groups

$$F=\frac{\text{Variance Between Groups}}{\text{Variance Within Groups}}$$

Bayesian Optimization (BO)

The BO code implementation was performed using the Bayesian Optimisation Python package [1]. Although BO allows for multi-objective optimization simultaneously, the PV parameters i.e. PCE, V_{oc} and J_{sc} were optimized independently in this study [2]. This choice was driven by the limited number of available experimental data points relative to the five-dimensional search space problem (5 electrolyte components), that could compromise the model reliability in a joint optimization framework. To evaluate the reliability of the Bayesian optimization surrogate before experimental validation, we applied a leave-one-out cross-validation (LOOCV) procedure independently to J_{sc}, V_{oc} and PCE. This strategy was selected because only 25 electrolyte formulations were available in a five-dimensional compositional space, so LOOCV maximizes the amount of training data while still probing generalization capability. The predictive performance followed the order Jsc > Voc > PCE, with R^2 values of 0.85, 0.69 and 0.49, respectively. This hierarchy can be rationalized from both physical and statistical viewpoints. J_{sc} is

more directly controlled by electrolyte composition and therefore follows a smoother composition–property relationship. V_{oc} spans a narrower dynamic range and is more sensitive to small variations in recombination-related processes, so even small absolute prediction errors result in a noticeable reduction in R^2. PCE is the most difficult target because it is a compound metric that depends on J_{sc}, V_{oc} and FF, while FF is not explicitly encoded in the composition vector and introduces additional device-level variability.

These limitations, together with the deliberate expansion of the BO search bounds beyond the initial DoE domain, likely explain why the closed-loop optimization did not surpass the best PCE already identified in the initial screening. Nevertheless, the BO/ML workflow successfully identified a non-intuitive low-iodide region leading to the highest V_{oc} obtained in this work.

The code we have used to carry out the BO can be found in the following repository: (https://github.com/RivasAntonio/ML-Assisted-Iodide-based-Electrolyte-Optimisation-and-Photoanode-Architecture-for-DSSC)

The overall workflow used to combine the reduced DoE screening, machine-learning analysis and Bayesian optimization is summarized in the scheme below.

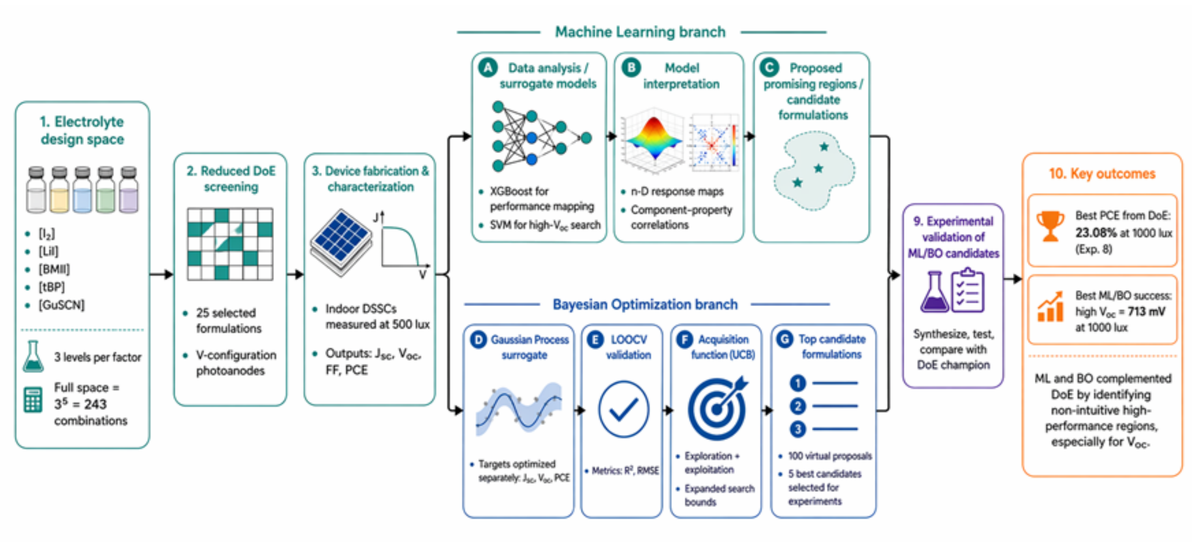

Figures S9–S11 compare the experimentally measured DoE dataset with the BO-proposed candidate formulations for J_{sc}, V_{oc} and PCE, respectively.

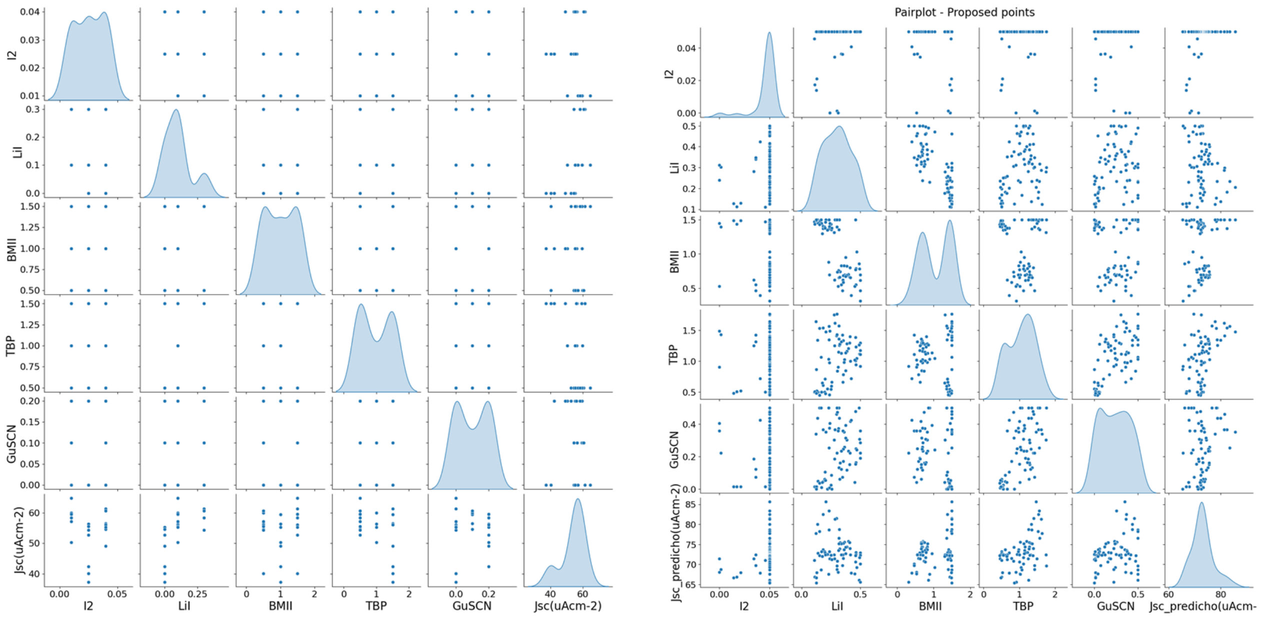

Fig. S9: Pairplot of the experimentally measured DoE dataset (left) and pairplot of BO-proposed candidate formulations with predicted Jsc values (right).

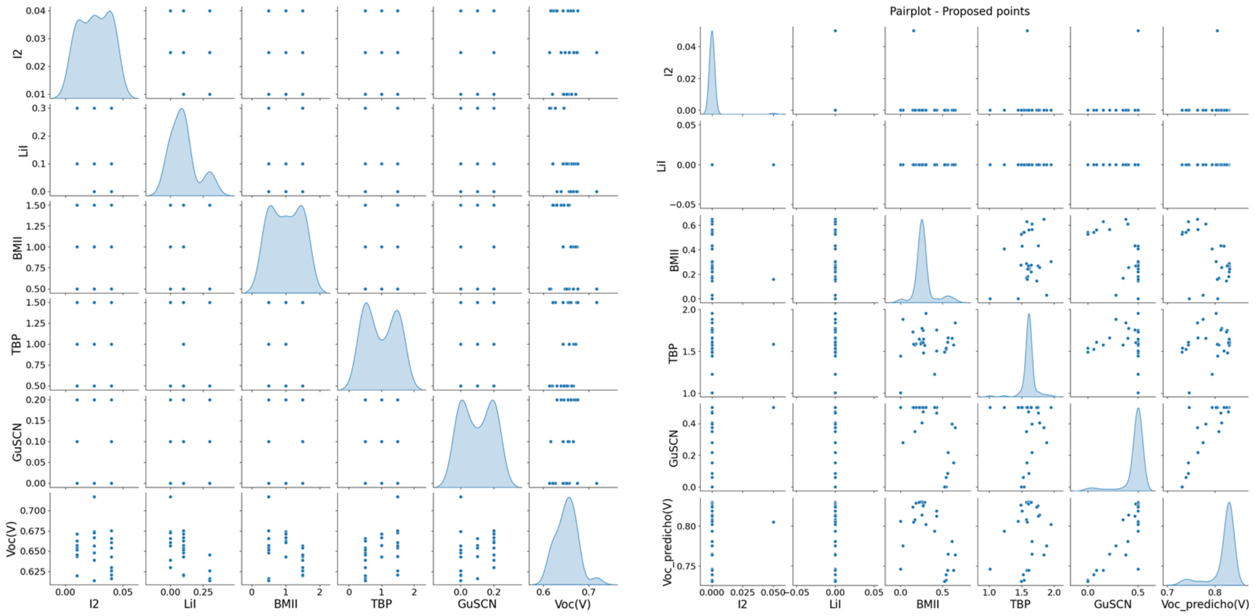

Fig. S10: Pairplot of the experimentally measured DoE dataset (left) and pairplot of BO-proposed candidate formulations with predicted Voc values (right).

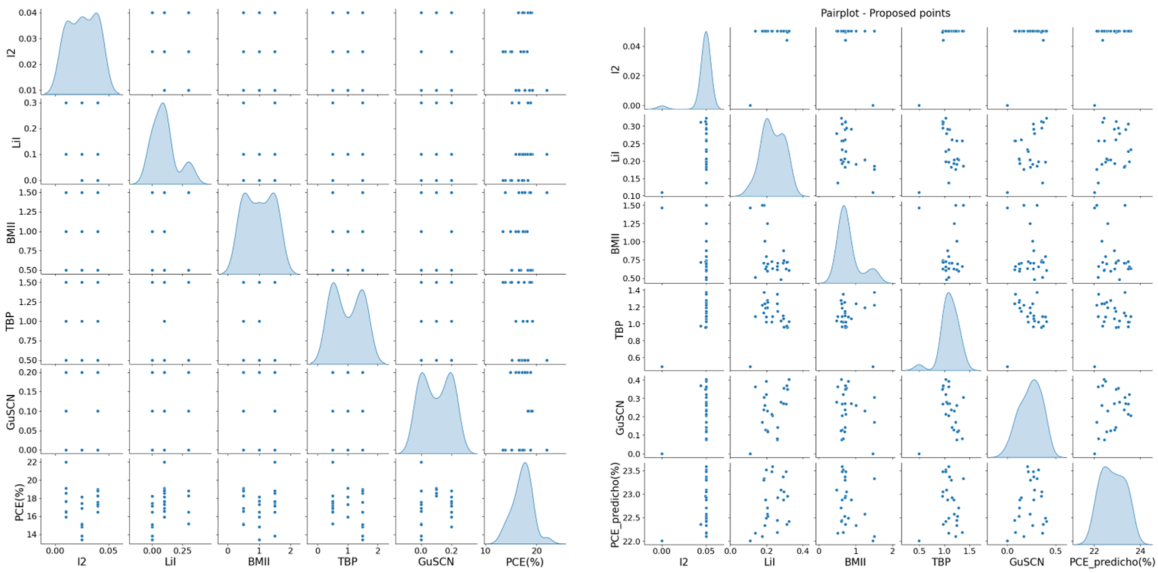

Fig. S11: Pairplot of the experimentally measured DoE dataset (left) and pairplot of BO-proposed candidate formulations with predicted PCE values (right).

The five top-ranked BO candidate formulations predicted for each target property are as listed in Table S3.

Table S3: Showing Bayesian Optimisation model predicted PV parameters of a) the power conversion efficiency, b) current density and c) the photovoltage.

**a)**	**molar concentrations**						
Experiment	[I_2]	[LiI]	[BMII]	[*t*BP]	[GuSCN]	PCE_predicted(%)	Improvement(%)
1	0.05	0.23	0.63	1.09	0.21	23.58	1.59
2	0.05	0.20	0.65	1.02	0.32	23.51	1.52
3	0.05	0.23	0.72	1.02	0.21	23.47	1.48
4	0.05	0.31	0.62	0.97	0.27	23.47	1.48
5	0.05	0.29	0.70	1.10	0.27	23.36	1.37

**b)**	**molar concentrations**						
Experiment	[I_2]	[LiI]	[BMII]	[*t*BP]	[GuSCN]	J_{sc} predicted (μA.cm^{-2})	Improvement (μA.cm^{-2})
1	0.05	0.21	1.50	1.47	0.35	85.61	20.85
2	0.05	0.23	1.50	1.56	0.25	83.38	18.62
3	0.05	0.18	1.37	1.54	0.37	82.44	17.67
4	0.05	0.30	1.50	1.41	0.46	81.52	16.76
5	0.05	0.29	1.50	1.61	0.37	81.32	16.55

**c)**	**molar concentrations**						
Experiment	[I_2]	[LiI]	[BMII]	[*t*BP]	[GuSCN]	V_{oc} predicted (V)	Improvement (V)
1	0	0	0.26	1.61	0.50	0.83	0.11
2	0	0	0.26	1.61	0.50	0.83	0.11
3	0	0	0.26	1.61	0.50	0.83	0.11
4	0	0	0.25	1.62	0.50	0.83	0.11
5	0	0	0.25	1.62	0.50	0.83	0.11

To further examine the high-Voc formulation predicted by ML/BO, UV–Vis absorption spectra of the V_{OC}- and J_{sc}-optimized electrolytes were recorded and are shown in Fig. S12.

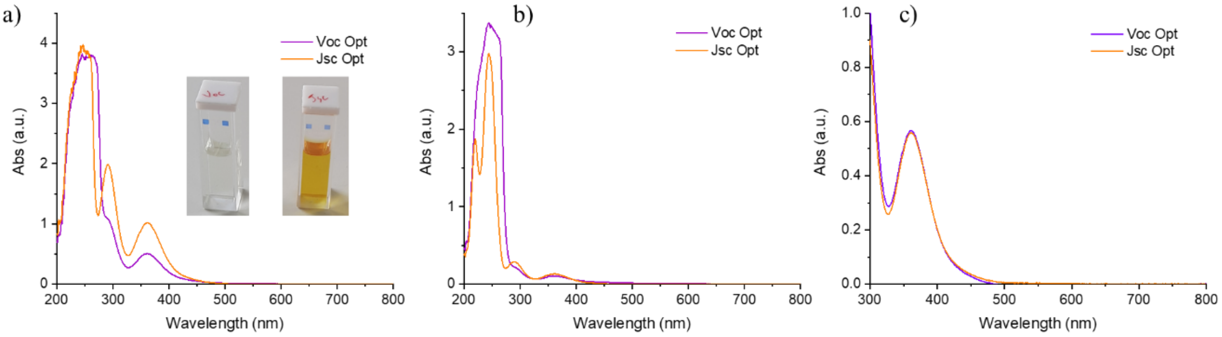

Fig. S12: The UV-Vis absorption spectra of the Machine Learning optimum predictions for the Voc and Jsc as in Tables S3 c) and S3 b), respectively, (Experiment 1) for both parameters. a) Inset, images of a 60-times dilution of the original electrolyte solutions, b) further dilutions, 3 times for Voc opt electrolyte and >30 times more for Jsc opt electrolyte solution, and c) comparison of the triiodide maximum absorption peak at 360 nm.

Electrolyte composition 0.01 M I_2, 0.1 M LiI, ~~1.5 M BMII~~ and 0.5 M *t*BP

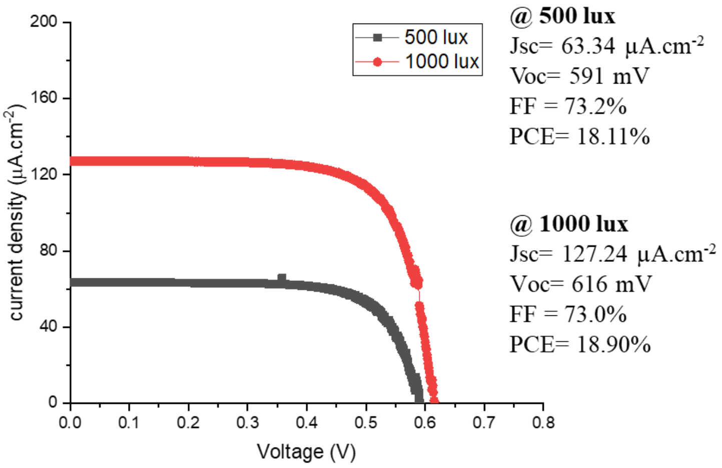

*Fig. S13: Current-voltage characteristics of **RK1**-based device using modified Electrolyte 8 composition (without BMII) at 500 and 1000 lux intensities.*

References

[1] F. Nogueira, «Bayesian Optimization: Open source constrained global optimization tool for Python.,» 2014.

[2] P. I. Frazier, «A Tutorial on Bayesian Optimization,» *arXiv:1807.02811,* 2018.
